# Opportunistic screening for incident cardiometabolic disease in metabolically healthy non-obese individuals: a prospective cohort study

**DOI:** 10.1186/s12933-026-03265-2

**Published:** 2026-07-01

**Authors:** Balázs Bogner, Matthias Jung, Marco Reisert, Juliane Maushagen, Susanne Rospleszcz, Thomas Kroencke, Tobias Pischon, Jeanette Schulz-Menger, Thoralf Niendorf, Henry Völzke, Christopher L. Schlett, Fabian Bamberg, Jana Taron, Jakob Weiss

**Affiliations:** 1 Department of Diagnostic and Interventional Radiology, University Medical Center Freiburg, Faculty of Medicine, University of Freiburg, Hugstetter Strasse 55, 79106 Freiburg, Germany; 2 Faculty of Medicine, Berta-Ottenstein-Programme, University of Freiburg, Freiburg, Germany; 3 Division of Medical Physics, Department of Diagnostic and Interventional Radiology, University Medical Center Freiburg, Faculty of Medicine, University of Freiburg, 79106 Freiburg, Germany; 4 Department of Stereotactic and Functional Neurosurgery, University Medical Center Freiburg, Faculty of Medicine, University of Freiburg, 79106 Freiburg, Germany; 5 Department of Diagnostic and Interventional Radiology, University Hospital Augsburg, Stenglinstr. 2, 86156 Augsburg, Germany; 6 Berlin Ultrahigh Field Facility (B.U.F.F.), Max Delbrück Center for Molecular Medicine in the Helmholtz Association, Berlin, Germany; 7 ECRC Experimental and Clinical Research Center, Corporate Member of Freie Universität Berlin Und Humbolt-Universität Berlin, Charité - Universitätsmedizin Berlin, Berlin, Germany; 8 Institute for Community Medicine, University Medicine Greifswald, Greifswald, Germany

**Keywords:** Magnetic resonance imaging, Metabolism, Obesity, Diabetes, Cardiovascular diseases

## Abstract

**Background:**

Metabolically healthy non-obese (MHN) individuals are considered at low cardiometabolic risk, yet a subset may harbor unfavorable visceral adiposity not captured by conventional anthropometric measures, including waist circumference (WC) and BMI.

**Methods:**

We conducted a prospective cohort study of 22,040 UK Biobank participants (median follow-up 4.2 years [interquartile range 3.4–5.6]) defined as MHN (BMI < 30 kg/m^2^, absence of diabetes or concurrent hypertension and hyperlipidemia). Visceral (VAT) and subcutaneous adipose tissue (SAT) volumes were quantified from whole-body MRI using a validated deep learning framework. Sex-specific VAT/SAT ratio cutoffs were derived from the German National Cohort based on prevalent cardiometabolic disease and applied to the UK Biobank. The primary outcome was incident major adverse cardiovascular events (MACE); the secondary outcome was incident type 2 diabetes. Categorical net reclassification improvement (NRI), quantifying the net proportion of individuals correctly reclassified between predefined risk categories, compared VAT/SAT ratio versus WC as competing classification approaches. Cox proportional hazards models assessed associations with outcomes after stepwise adjustment for age, sex, smoking, WC, and BMI. Nested models with and without VAT/SAT ratio were compared to test for added value beyond other factors.

**Results:**

The VAT/SAT ratio improved risk classification over WC for MACE (NRI 0.088, 95%CI 0.019–0.158, *p* = 0.013) and diabetes (NRI 0.102, 95% CI 0.024–0.181, *p* = 0.010). High VAT/SAT ratio independently predicted MACE (adjusted hazard ratio [aHR] 1.30, 95%CI 1.02–1.66, *p* = 0.037) and diabetes (aHR 1.77, 95% CI 1.34–2.33, *p* < 0.001) after full adjustment. Adding VAT/SAT to fully adjusted models improved discrimination for MACE (C-index 0.694 vs. 0.690, *p* = 0.036) and diabetes (C-index 0.723 vs. 0.715, *p* < 0.001).

**Conclusion:**

The VAT/SAT ratio identifies MHN individuals at elevated cardiometabolic risk beyond conventional anthropometric measures, with particularly strong associations for incident diabetes. These findings support the concept of opportunistic imaging-based risk assessment and provide the prognostic foundation for future trials investigating whether targeted intervention in VAT/SAT-reclassified individuals improves outcomes.

**Supplementary Information:**

The online version contains supplementary material available at 10.1186/s12933-026-03265-2.

## Research insights

**What is currently known about this topic?**

Visceral (VAT) and subcutaneous fat (SAT) compartments have distinct metabolic characteristics. BMI and waist circumference (WC) cannot distinguish between these fat compartments. Outcome data on VAT/SAT ratio in metabolically healthy non-obese (MHN) individuals are lacking.

**What is the key research question?**

Does MRI-derived VAT/SAT ratio identify cardiometabolic risk beyond BMI and WC in MHN individuals?

**What is new?**

VAT/SAT ratio provided incremental prognostic value beyond traditional anthropometric measures. VAT/SAT ratio reclassified individuals missed by WC, especially for diabetes.

**How might this study influence clinical practice?**

MRI-based VAT/SAT quantification could enhance opportunistic cardiometabolic risk assessment.

## Background

Cardiometabolic diseases, including type 2 diabetes and cardiovascular disease, remain leading causes of global morbidity and mortality [1, 2]. Body mass index (BMI) and waist circumference (WC) are widely used to assess adiposity and cardiometabolic risk, but provide limited information on body fat distribution and may not reflect variations in risk across different body composition phenotypes [3, 4].

Magnetic resonance imaging (MRI) can directly quantify visceral adipose tissue (VAT) and subcutaneous adipose tissue (SAT) [5]. VAT is metabolically active, secreting pro-inflammatory cytokines and free fatty acids via portal drainage, and is strongly associated with cardiometabolic risk. SAT, while also metabolically active, has distinct and comparatively less pathogenic properties, including adiponectin secretion and lipid buffering capacity [6, 7]. Given these contrasting metabolic profiles, the VAT/SAT ratio has emerged as a promising imaging biomarker reflecting the balance between these fat compartments, with established associations between elevated VAT/SAT ratios and cardiometabolic risk factors independent of total adiposity [8–10].

However, large-scale prospective population-based studies investigating the association between VAT/SAT ratio and incident cardiometabolic outcomes remain limited [11]. In addition, a critical knowledge gap exists regarding apparently healthy individuals with normal BMI and absent metabolic risk factors but excess VAT accumulation, a population that may harbor increased cardiometabolic risk not captured by conventional anthropometric measures such as WC [3]. Understanding whether the VAT/SAT ratio can identify high-risk individuals within this traditionally overlooked population could improve early risk stratification and targeted prevention strategies.

Therefore, we investigated the association of MRI-derived VAT/SAT ratio with incident major adverse cardiovascular events (MACE) and diabetes in metabolically healthy non-obese (MHN) individuals from the UK Biobank (UKB), hypothesizing that an elevated VAT/SAT ratio can identify individuals at risk beyond traditional risk factors, including BMI and WC, in this apparently low-risk population.

## Methods

### Study overview

**Fig. 1:**
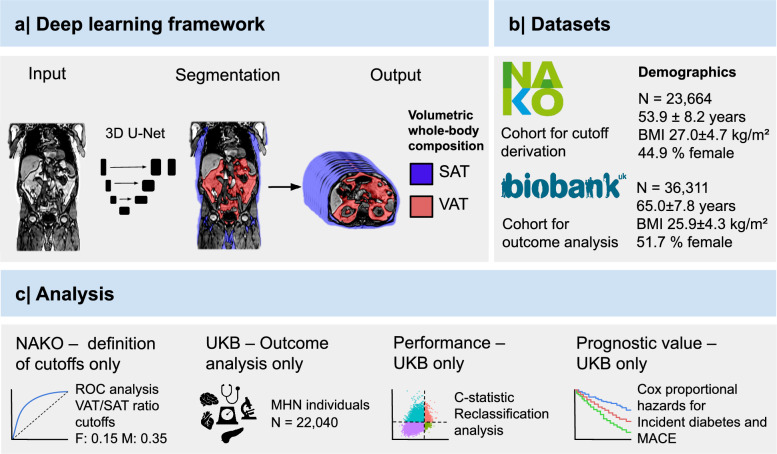
Study Overview. **a** Deep learning framework utilizing 3D U-Net for whole-body MRI segmentation to quantify volumetric SAT and VAT. **b** Two cohorts were analyzed: NAKO (N = 23,664) for cutoff derivation and UKB (N = 36,311) with longitudinal follow-up data. (**c**) Study workflow: Sex-specific VAT/SAT ratio cutoffs were derived from NAKO using ROC analysis based on prevalent cardiometabolic disease, then applied to UKB MHN individuals (N = 22,040; subset of the full UKB cohort after applying metabolic health and obesity criteria) to assess reclassification performance and prognostic value for incident MACE and diabetes. BMI, body mass index; F, female; M, male; MACE, major adverse cardiovascular events; MHN, metabolically healthy non-obese; MRI, magnetic resonance imaging; NAKO, German National Cohort; ROC, receiver operating characteristic; SAT, subcutaneous adipose tissue; UKB, UK Biobank; VAT, visceral adipose tissue

The primary aim was to assess whether the MRI-derived VAT/SAT ratio can be used as an opportunistic imaging biomarker to identify individuals at increased risk for incident MACE and diabetes in a cohort of MHN individuals of the UKB. Adipose tissue volumes were quantified from whole-body MRI using a validated deep learning framework [5]. First, sex-specific VAT/SAT ratio cutoffs were derived in the German National Cohort (NAKO) study based on prevalent cardiometabolic disease to reduce overfitting. These cutoffs were then applied to the UKB to investigate risk reclassification for incident MACE and diabetes for VAT/SAT ratio *vs.* WC, a common metric to assess adiposity in daily practice. In addition, the association between VAT/SAT ratio and outcomes was assessed via univariable and multivariable Cox proportional hazard regression analyses adjusted for traditional cardiometabolic risk factors. An overview of the study design is demonstrated in Fig. 1**.**

### Data sources

#### UK Biobank

The UK Biobank is a prospective population-based study of over 500,000 volunteers aged 38–73 years that provides detailed clinical parameters and outcome data [12, 13]. During baseline assessment between 2006 and 2010, extensive questionnaire data, physical measurements, and biological samples were collected. The current analysis included participants from the multimodal imaging sub-cohort who underwent T1-weighted 3D two-point VIBE Dixon MRI (1.5 T MAGNETOM Aera, Siemens Healthineers) between 2014 and 2022 [14].

#### Metabolic health and obesity

**Fig. 2:**
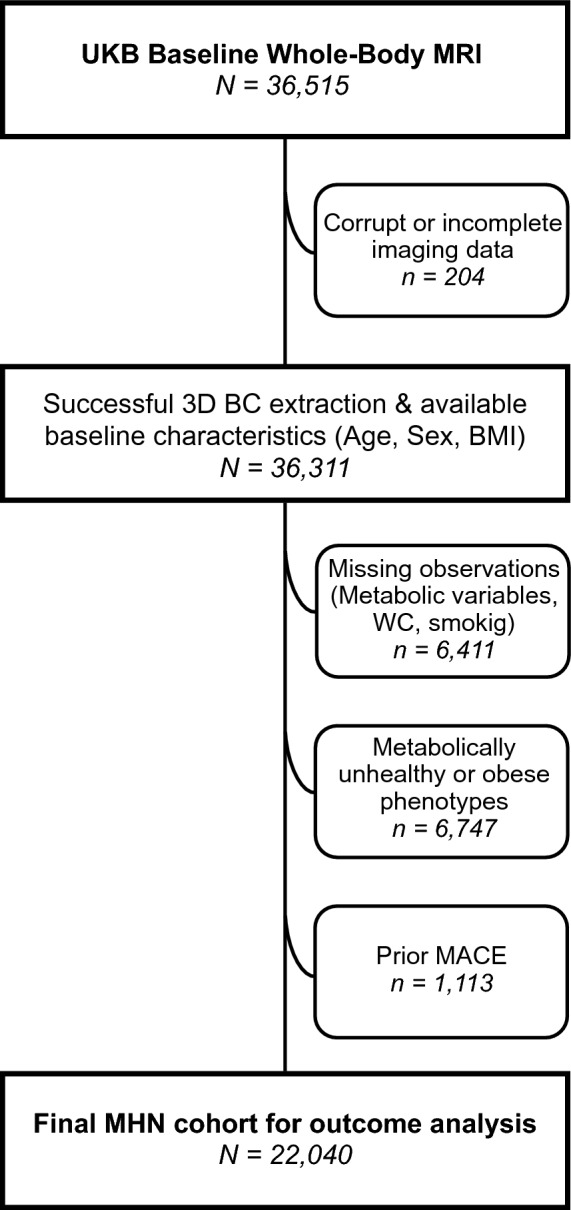
Consort diagram. Flowchart demonstrating participant selection and exclusion criteria for the UKB cohort. 3D BC, three-dimensional body composition; BMI, body mass index; MACE, major adverse cardiovascular events; MRI, magnetic resonance imaging; MHN, metabolically healthy non-obese; NAKO, German National Cohort; UKB, UK Biobank; SAT subcutaneous adipose tissue; VAT, visceral adipose tissue; WC, waist circumference

UKB participants were classified into metabolic phenotypes based on obesity and metabolic health status. Height (in m) and weight (in kg) were recorded during the imaging visit. BMI was calculated as weight divided by height squared (in m^2^). Individuals with a BMI ≥ 30 kg/m^2^ were categorized as obese, per World Health Organization (WHO) criteria [3]. Participants were classified as metabolically unhealthy if they had diabetes or a combination of hypertension and hyperlipidemia [15]. Diabetes (International Classification of Diseases [ICD]-10 codes E10–E14; ICD-9 code 250) and hypertension (ICD-10 codes I10–I15; ICD-9 codes 401–405) status were derived from ICD records up to and including the imaging visit. Hyperlipidemia was defined as triglycerides ≥ 150 mg/dL, total cholesterol ≥ 5.18 mmol/L, LDL ≥ 3.37 mmol/L, or HDL < 1.04 mmol/L in men and < 1.30 mmol/L in women. Serum lipid markers were available from the baseline assessment only. This study focused on MHN individuals, defined as participants with a BMI < 30 kg/m^2^ (underweight [< 18.5 kg/m^2^], normal weight [18.5–24.9 kg/m^2^], and overweight [25–29.9 kg/m^2^]) without diabetes or the combination of hypertension and hyperlipidemia. After excluding participants with incomplete imaging data (n = 204), missing clinical covariates (n = 6,411), prior MACE (n = 1,113), and metabolically unhealthy or obese phenotypes (n = 6,747), the final cohort comprised 22,040 MHN individuals (Fig. 2a).

#### German National Cohort (NAKO)

The NAKO is an ongoing cohort study of 200,000 participants aged 20–72 years investigating common diseases across 18 sites in Germany [16]. NAKO data were used exclusively for sex-specific VAT/SAT ratio cutoff derivation and were not included in outcome analyses. Detailed information on the NAKO cohort, including imaging protocols and exclusion criteria, is provided in the Supplemental Material (Supplemental Methods 1, Supplemental Fig. 1).

#### Body composition analysis

VAT and SAT volumes were automatically quantified from whole-body T1-weighted Dixon MRI using a validated deep learning framework [5]. VAT/SAT ratios were computed as the quotient of total VAT to SAT volumes (in dm^3^) for each participant. To avoid overfitting, sex-specific VAT/SAT ratio cutoffs were derived in NAKO participants only using receiver operating characteristic (ROC) curve analysis based on prevalent cardiometabolic disease. This analysis yielded optimal thresholds of 0.356 for MACE and 0.398 for diabetes in males, and 0.140 for MACE and 0.142 for diabetes in females, respectively. To facilitate clinical utility and maintain consistency across outcomes, we chose unified sex-specific cutoffs of 0.35 for males and 0.15 for females, which were applied to classify UKB participants as having "high" or "low" VAT/SAT ratios for all subsequent analyses.

#### Clinical covariates

The following a priori defined risk factors were included in multivariable analyses: age (in years), sex, smoking status, WC (in cm), and BMI (in kg/m^2^). Smoking status and WC were assessed at the imaging visit. Smoking was categorized as ever (current or former) or never smoker. For reclassification analysis, WC cutoffs were defined according to the WHO guidelines as > 94 cm for men and > 80 cm for women to identify increased metabolic risk [17].

#### Endpoints

The primary endpoint was MACE defined as myocardial infarction (ICD-10 codes I21–I22), ischemic stroke (ICD-10 code I63), or cardiovascular disease mortality (ICD-10 codes I00–I78) occurring within the follow-up period. The secondary endpoint was incident diabetes, defined as new diagnosis codes (ICD-10 codes E10–E14; ICD-9 code 250) occurring after the MRI scan date in participants without prior diabetes history. Primary care Read codes were not available for this analysis; outcome ascertainment was therefore based on hospital ICD records only. Follow-up time was defined as the period from the MRI scan date to the earliest of: occurrence of the outcome event, loss to follow-up, or administrative censoring (October 31, 2022 for ICD-coded outcomes; May 25, 2023 for mortality data).

#### Statistical analysis

Baseline characteristics are presented as mean ± standard deviation (SD) or median with interquartile ranges (IQR) for continuous variables and absolute counts with percentages for categorical variables. Normality assumptions for continuous variables were informally assessed using Q–Q plots and histograms due to the large sample sizes. Categorical net reclassification improvement (NRI) was calculated to quantify reclassification performance, using predefined binary risk categories based on established sex-specific WC cutoffs (> 94 cm for males, > 80 cm for females) and NAKO-derived VAT/SAT ratio thresholds (> 0.35 for males, > 0.15 for females), comparing VAT/SAT ratio and WC as competing classification approaches. Cox proportional hazards models assessed associations between VAT/SAT ratio and (1) incident MACE, as well as (2) diabetes, using a stepwise adjustment approach: Model 1 unadjusted; Model 2 adjusted for age, sex, and smoking status; Models 3 and 4 added WC and BMI respectively to assess their individual contributions; Model 5 (fully adjusted) included age, sex, smoking status, WC, and BMI. The proportional hazards assumption was verified using scaled Schoenfeld residuals. To assess incremental prognostic value, nested Cox proportional hazards models with and without VAT/SAT ratio were compared using likelihood ratio tests and Harrell’s C-index. Results are reported as hazard ratios (HR) and adjusted hazard ratios (aHR) with 95% confidence intervals (CI).

To ensure the robustness of the primary findings, sensitivity analyses were performed. These included analysis of the VAT/SAT ratio as a continuous predictor, exclusion of participants who experienced an event within the first one or two years of follow-up, restriction to participants with a minimum follow-up of one or two years, and Fine & Gray competing-risk models. Primary analyses were additionally repeated after imputation of missing covariates and with an enhanced MHN definition incorporating medication use and biochemical markers. Further exploratory analyses included sex- and age-stratified models, analyses across all four metabolic phenotypes, and comparisons with alternative anthropometric indices including waist-to-hip ratio and waist-to-height ratio. Full results are provided in the Supplemental Material. Statistical significance was set at *p* < 0.05. All analyses were performed using R statistical software (version 4.3.0; R Core Team, https://www.r-project.org/).

## Results

### Study cohort

The final cohort comprised 22,040 MHN UKB participants (mean age 64.6 ± 7.8 years, 53.3% female). 12,656 (57.4%) had a low and 9,384 (42.6%) a high VAT/SAT ratio stratified by sex-specific cutoffs. Participants with high VAT/SAT ratios were predominantly male (55.7% *vs.* 40.0%), older (66.0 ± 7.4 *vs.* 63.5 ± 7.9 years), had higher BMI (25.4 ± 2.5 *vs.* 23.7 ± 2.8 kg/m^2^), larger WC (89.5 ± 9.1 vs. 81.5 ± 10.1 cm), and were more frequently current or former smokers (42.1% *vs.* 36.4%). Detailed baseline characteristics of the UKB cohort are presented in Table 1.

**Table 1:** Baseline characteristics stratified by VAT/SAT ratio in the UKB cohort

	Overall (N = 22,040)	Low VAT/SAT (N = 12,656)	High VAT/SAT (N = 9,384)
Age (Years)	64.6 ± 7.8	63.5 ± 7.9	66.0 ± 7.4
Female sex	11,749 (53.3%)	7588 (60.0%)	4161 (44.3%)
BMI (kg/m^2^)	24.4 ± 2.8	23.7 ± 2.8	25.4 ± 2.5
WC (cm)	84.9 ± 10.4	81.5 ± 10.1	89.5 ± 9.1
Current/former smoker	8559 (38.8%)	4605 (36.4%)	3954 (42.1%)
SAT (dm^3^)	13.85 (IQR 11.01–17.16)	13.43 (IQR 10.37–16.87)	14.41 (IQR 11.76–17.49)
VAT (dm^3^)	2.93 (IQR 1.71–4.55)	1.93 (IQR 1.19–2.92)	4.51 (IQR 3.35–5.97)
VAT/SAT ratio	0.20 (IQR 0.12–0.34)	0.13 (IQR 0.09–0.25)	0.37 (IQR 0.19–0.44)

Values are presented as mean ± SD for normally distributed continuous variables, median (IQR) for skewed continuous variables, and n (%) for categorical variables. BMI, body mass index; IQR, interquartile range; SAT, subcutaneous adipose tissue; SD, standard deviation; VAT, visceral adipose tissue; VAT/SAT ratio, visceral-to-subcutaneous adipose tissue ratio; WC, waist circumference

***NAKO cohort:*** Baseline characteristics of the NAKO cohort (n = 23,664), which was used to derive sex-specific VAT/SAT ratio cutoffs, are presented in the Supplemental Material (**Supplemental **Table 1).

### Distribution and reclassification of VAT/SAT ratio vs. WC

**Fig. 3:**
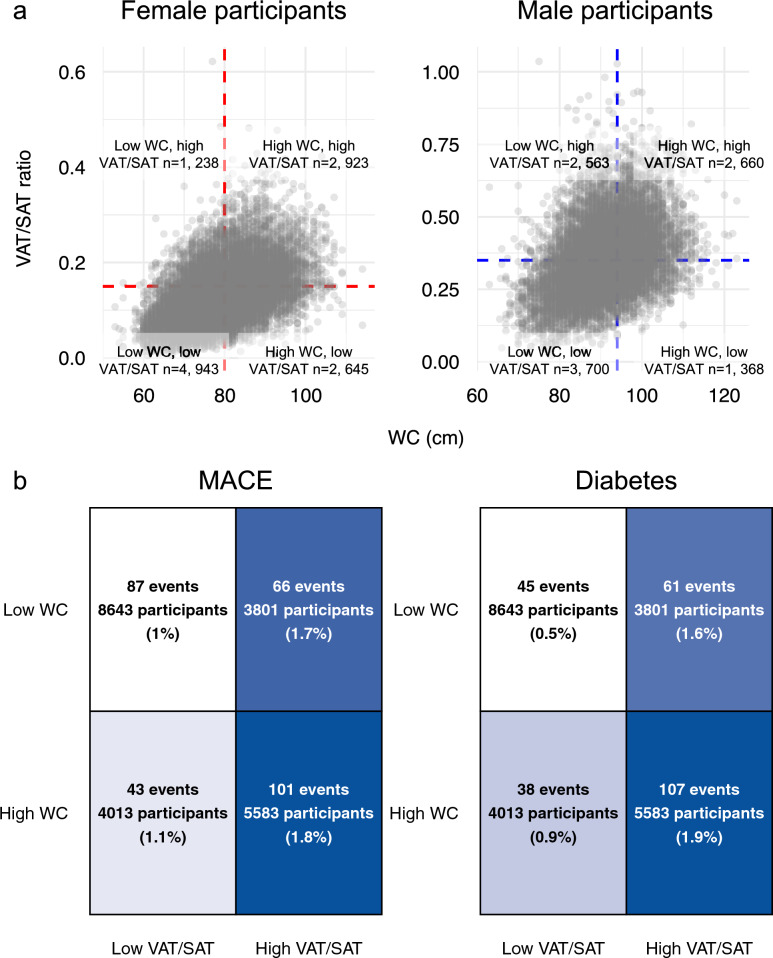
Distribution of VAT/SAT ratio and waist circumference, and reclassification analysis. **a** Scatter plots showing the distribution of participants by sex across four risk groups defined by sex-specific cutoffs for WC (80 cm for females, 94 cm for males, red and blue vertical dashed lines) and VAT/SAT ratio (0.15 for females, 0.35 for males, red and blue horizontal dashed lines). Each point represents one participant. **b** Risk stratification matrices displaying event rates for incident MACE and diabetes across the four risk groups. Numbers indicate events, total participants, and event rates (%) in each cell. Color intensity reflects event rate magnitude (white to dark blue gradient). MACE, major adverse cardiovascular events; SAT, subcutaneous adipose tissue; VAT, visceral adipose tissue; WC, waist circumference

Based on established sex-specific WC cutoffs (80 cm for females, 94 cm for males) and NAKO-derived VAT/SAT ratio thresholds (0.15 for females, 0.35 for males), participants were classified into four distinct risk categories (Fig. 3a**):** low WC with low VAT/SAT (females: n = 4,943, 43.8%; males: n = 3,700, 33.8%), low WC with high VAT/SAT (females: n = 1,238, 11.0%; males: n = 2,563, 23.4%), high WC with low VAT/SAT (females: n = 2,645, 23.4%; males: n = 1,368, 12.5%), and high WC with high VAT/SAT (females: n = 2,923, 25.9%; males: n = 3,660, 33.4%). Notably, 11.0% of females and 23.4% of males with low WC exhibited high VAT/SAT ratios.

WC and VAT/SAT ratio classifications were concordant in 64.5% (14,226/22,040) and discordant in 35.5% (7,814/22,040) of participants. Event rates across the four risk groups for incident MACE and diabetes are shown in Fig. 3b. For MACE, the highest event rate was found in the high WC with high VAT/SAT group (1.8%), followed by low WC with high VAT/SAT (1.7%), high WC with low VAT/SAT (1.1%), and low WC with low VAT/SAT (1.0%). A similar pattern was observed for diabetes (1.9%, 1.6%, 0.9%, and 0.5%, respectively).

Among discordant cases (n = 7,814), VAT/SAT ratio demonstrated superior risk discrimination. For MACE, VAT/SAT up-classified 66 participants (0.8%, 1.7% event rate) and down-classified 43 participants (0.6%, 1.1% event rate), with an NRI of 0.088 (95% CI 0.019–0.158, *p* = 0.013). For diabetes, VAT/SAT up-classified 61 participants (0.8%, 1.6% event rate) and down-classified 38 participants (0.5%, 0.9% event rate), yielding an NRI of 0.102 (95% CI 0.024–0.181, *p* = 0.010).

### Association between VAT/SAT ratio and clinical outcomes

**Fig. 4:**
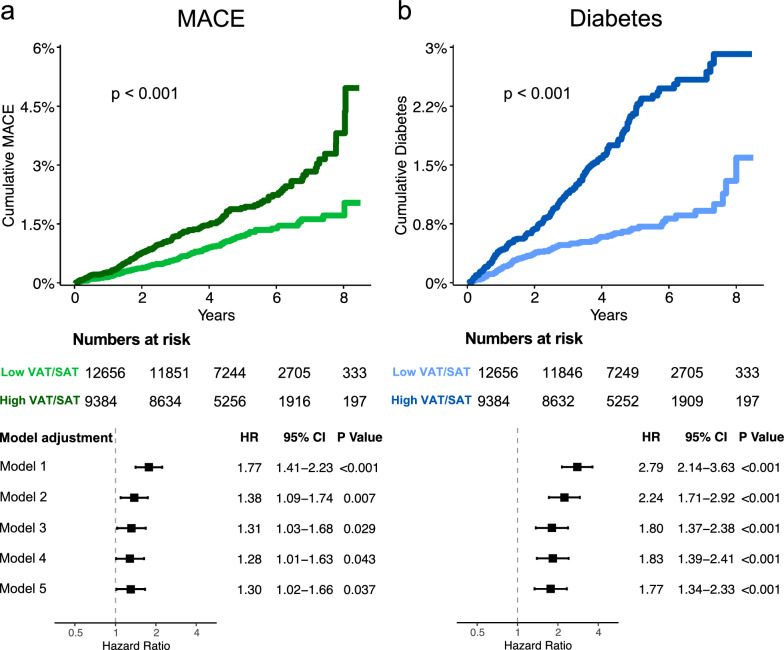
Associations of VAT/SAT ratio with incident MACE and diabetes in metabolically healthy non-obese individuals. **a − b** Cumulative incidence curves for MACE and diabetes stratified by VAT/SAT ratio groups (high *vs.* low based on sex-specific cutoffs) with numbers at risk shown below each plot. Forest plots display hazard ratios from Cox proportional hazards models with stepwise adjustment. Model 1: unadjusted; Model 2: adjusted for age, sex, and smoking status; Model 3: Model 2 + waist circumference; Model 4: Model 2 + BMI; Model 5: Model 2 + waist circumference + BMI. Log-rank *p* < 0.001 for both outcomes. BMI, body mass index; HR, hazard ratio; MACE, major adverse cardiovascular events; SAT, subcutaneous adipose tissue; VAT, visceral adipose tissue

**MACE:** Over a median follow-up of 4.2 years [IQR 3.4–5.6], 297 cases of MACE (1.35%) occurred. Cumulative incidence curves showed a significantly higher event rate in the high *vs.* low VAT/SAT group (2.5% *vs.* 1.6%, log-rank *p* < 0.001) (Fig. 4a).

Univariable Cox regression revealed an independent association between VAT/SAT ratio and MACE (HR: 1.77, 95% CI 1.41–2.23, *p* < 0.001). This association remained robust after stepwise adjustment for age, sex, and smoking status (aHR: 1.38, 95% CI 1.09–1.74, *p* = 0.007), and when additionally adjusting for either WC (aHR: 1.31, 95% CI 1.03–1.68, *p* = 0.029) or BMI (aHR: 1.28, 95% CI 1.01–1.63, *p* = 0.043) separately. In the fully adjusted model including all risk factors combined, VAT/SAT remained independently associated with MACE (aHR: 1.30, 95% CI 1.02–1.66, *p* = 0.037) (Fig. 4a).

**Diabetes:** Over a median follow-up of 4.2 years [IQR 3.4–5.6], 251 cases of incident diabetes (1.14%) occurred. Similar to MACE, cumulative incident curves showed significantly higher event rate in the high *vs.* low VAT/SAT group (1.8% *vs.* 0.7%, log-rank *p* < 0.001) (Fig. 4b).

Univariable Cox regression revealed an independent association between VAT/SAT ratio and diabetes (HR: 2.79, 95% CI 2.14–3.63, *p* < 0.001). This association remained robust after stepwise adjustment for age, sex, and smoking status (aHR: 2.24, 95% CI 1.71–2.92, *p* < 0.001), and when additionally adjusting for either WC (aHR: 1.80, 95% CI 1.37–2.38, p < 0.001) or BMI (aHR: 1.83, 95% CI 1.39–2.41, *p* < 0.001) separately. In the fully adjusted model including all risk factors combined, VAT/SAT ratio continued to independently predict diabetes (aHR: 1.77, 95% CI 1.34–2.33, *p* < 0.001) (Fig. 4b).

### Incremental prognostic value

To test whether VAT/SAT ratio adds incremental value to a baseline model with clinical risk factors, including age, sex, smoking, WC, and BMI only, nested Cox proportional hazard models were compared. Adding VAT/SAT ratio to the baseline model resulted in modest but significant improvement to predict MACE (C-index 0.694 *vs.* 0.690, *p* = 0.036) and diabetes (C-index 0.723 vs. 0.715, *p* < 0.001).

### Sensitivity analyses

Results for incident diabetes were largely consistent across sensitivity analyses and with the primary finding, though associations were attenuated in females and participants aged < 60 years. For incident MACE, results were more variable and attenuated in several analyses. Full results are provided in Supplemental Tables 3–9 and Supplemental Figs. 2–4.

## Discussion

In this prospective cohort study of 22,040 MHN individuals from the UKB, we demonstrated that MRI-derived VAT/SAT ratio provides independent prognostic value for incident MACE and diabetes beyond common and easily accessible demographic and anthropometric factors. We found that (1) after adjustment for age, sex, smoking status, BMI, and WC, high VAT/SAT ratio remained significantly associated with a 30% increased MACE risk, and a 77% increased diabetes risk, (2) the VAT/SAT ratio provided modest but statistically significant improvement in reclassification over WC-based risk categories for both outcomes, and (3) adding VAT/SAT ratio to a baseline model of demographic and anthropometric risk factors allowed for statistically significant incremental improvement in MACE and diabetes risk estimation in apparently healthy individuals from the general population.

The biological differences between VAT and SAT are well known, with the VAT/SAT ratio capturing the relative balance between metabolically adverse and comparatively benign fat compartments [11]. Cross-sectional studies consistently demonstrate associations between elevated VAT/SAT ratios and cardiometabolic risk factors [8–10, 18]. Prospective outcome data, however, remain limited: existing cohort studies examining incident cardiovascular events or diabetes are few, predominantly small, and largely derived from selected clinical populations such as cancer screening programs or patients with established coronary artery disease undergoing revascularization [19–23]. Our findings address this gap, demonstrating that the MRI-derived VAT/SAT ratio independently predicts both incident MACE and diabetes in a large population-based cohort of MHN individuals, a population traditionally considered low-risk in whom excess visceral adiposity often goes undetected by conventional anthropometric measures.

We observed stronger associations between VAT/SAT ratio and incident diabetes compared to MACE. This may reflect the ability of the VAT/SAT ratio to capture the fundamental contrast between VAT, which drives hepatic insulin resistance through portal delivery of free fatty acids and pro-inflammatory mediators, and SAT, which provides protective adiponectin secretion and lipid buffering [6, 7]. In contrast, cardiovascular events result from complex, multifactorial processes involving endothelial dysfunction, atherosclerosis progression, and thrombotic mechanisms that develop over longer timeframes through multiple intermediate pathways [24, 25]. This pattern was further supported by sensitivity analyses, in which the diabetes association remained robust while the MACE association was attenuated, likely reflecting low event rates in this low-risk population over a relatively short median follow-up of 4.2 years. Longer follow-up will be needed to confirm the stability of MACE effect estimates.

Importantly, while most studies investigating metabolic phenotypes have used MHN individuals as a reference group for comparison with metabolically unhealthy populations, comprehensive analysis of risk stratification within this traditionally low-risk group has been limited [15, 26, 27]. Our findings suggest that meaningful risk heterogeneity exists even within the MHN phenotype, which can be captured by automated analysis of medical imaging studies. In exploratory analyses across metabolic phenotypes, the association between VAT/SAT ratio and incident diabetes was significant in MHN, metabolically unhealthy non-obese, and metabolically unhealthy obese, but not in metabolically healthy obese individuals. This may reflect the metabolically protective role of SAT in healthy obesity [28]. For MACE, significant associations were limited to the MHN group, likely reflecting reduced statistical power in smaller subgroups rather than true effect modification.

BMI and WC are well-established, easily obtainable measures of total and abdominal adiposity, but neither allows assessment of relative VAT and SAT proportions [3, 4]. Our NRI analysis demonstrated that the VAT/SAT ratio provided statistically significant reclassification improvements for both MACE and diabetes compared to WC, and nested model comparisons confirmed incremental prognostic value beyond WC and BMI. However, the magnitude of these improvements was modest. Therefore, the VAT/SAT ratio should be viewed as a complementary tool rather than a replacement for established anthropometric measures. Whole-body MRI is not performed in routine clinical practice, and we do not recommend specifically ordering it for body composition assessment. A more pragmatic approach would be to use an opportunistic strategy where VAT and SAT volumes are automatically extracted from MRI or CT scans performed for routine clinical indications, such as abdominal imaging for hepatic or renal assessment or oncological follow-up [29]. Deep learning-based segmentation methods are increasingly enabling large-scale automated quantification of body composition from routine scans [5, 30]. If this approach is validated in broader clinical cohorts, it could represent a realistic pathway towards clinical implementation, providing prognostic information beyond established risk estimates, at no additional cost and with no disruption to existing workflows.

This study has several limitations. First, the UKB cohort is predominantly white adults older than 45 years, and the findings may not be generalizable to more diverse populations [31]. Second, data on diet, physical activity, and socioeconomic status were unavailable for most participants and may have introduced residual confounding. Third, serum lipid markers used to define hyperlipidemia were only available from the baseline assessment (2006–2010), introducing a potential temporal gap in metabolic health classification at the imaging visit (2014–2022). As diabetes and hypertension were derived from ICD records up to the imaging visit, any misclassification of MHN status was limited to participants who developed hyperlipidemia between the baseline and imaging visits and who also had concurrent hypertension on ICD records. While the magnitude and direction of this misclassification cannot be precisely quantified, the number of affected participants is expected to be small, and the overall impact on study results is likely limited. Fourth, the VAT/SAT ratio cutoffs were derived from NAKO participants with prevalent cardiometabolic disease and, given differences in age and body composition, may not be fully transferable to the UKB population. Nevertheless, analyzing the VAT/SAT ratio as a continuous variable confirmed independent associations, underscoring the robustness of our findings. Finally, independent external validation of the outcome analyses is lacking. Although the cross-cohort design, with cutoff derivation in NAKO and outcome analyses in UKB, reduces overfitting, it does not constitute formal external validation. Replication in more diverse, independent cohorts is needed before broader clinical implementation.

## Conclusions

The MRI-derived VAT/SAT ratio independently predicts incident MACE and diabetes beyond WC and BMI in metabolically healthy non-obese individuals, with especially strong associations for diabetes. The VAT/SAT ratio provided statistically significant reclassification improvement over WC-based risk categories. These findings support the potential of MRI-based body composition analysis for personalised cardiometabolic risk assessment in apparently healthy individuals and lay the groundwork for trials testing whether VAT/SAT-guided interventions improve outcomes.

## Supplementary Information

Additional file1 (DOCX 7273 kb)

## Data Availability

The data that support the findings of this study are available from UK Biobank (https://www.ukbiobank.ac.uk/enable-your-research/apply-for-access) but restrictions apply to the availability of these data, which were used under license for the current study under Application Number 80337, and so are not publicly available. Researchers may apply directly to UK Biobank for access.

## Abbreviations

aHR: Adjusted hazard ratio
BMI: Body mass index
CI: Confidence interval
HR: Hazard ratio
ICD: International classification of diseases
IQR: Interquartile range
MACE: Major adverse cardiovascular events
MHN: Metabolically healthy non-obese
MRI: Magnetic resonance imaging
NAKO: German National Cohort
NRI: Net reclassification improvement
ROC: Receiver operating characteristic
SAT: Subcutaneous adipose tissue
SD: Standard deviation
UKB: UK biobank
VAT: Visceral adipose tissue
WC: Waist circumference
WHO: World Health Organization
