# Supplementary material for: Opportunistic screening for incident cardiometabolic disease in metabolically healthy non-obese individuals: a prospective cohort study
Source: Cardiovasc Diabetol. 2026 Jul 1;25:190. doi: 10.1186/s12933-026-03265-2 (PMC13330449; doi:10.1186/s12933-026-03265-2)
Supplement: Supplementary file 1 — Additional file1 (DOCX 7273 kb) [file 12933_2026_3265_MOESM1_ESM.docx]

**Opportunistic screening for incident cardiometabolic disease in metabolically healthy non-obese individuals: a prospective cohort study**

Balázs Bogner, Matthias Jung, Marco Reisert, Juliane Maushagen, Susanne Rospleszcz, Thomas Kroencke, Tobias Pischon, Jeanette Schulz-Menger, Thoralf Niendorf, Henry Völzke, Christopher Schlett, Fabian Bamberg, Jana Taron, Jakob Weiss

I SUPPLEMENTAL METHODS 2

II SUPPLEMENTAL RESULTS 5

III SUPPLEMENTAL TABLES 8

IV SUPPLEMENTAL FIGURES 14

V SUPPLEMENTAL REFERENCES 18

## I SUPPLEMENTAL METHODS

**Data sources**

***German National Cohort (NAKO)***

The NAKO represents a large-scale, interdisciplinary prospective cohort study designed to investigate the etiology of major chronic diseases, including cardiovascular disease, diabetes, and cancer. The study enrolled 200,000 generally healthy participants aged 20–72 years across 18 study centers throughout Germany [1]. A subset of over 30,000 participants underwent comprehensive whole-body MRI at five designated imaging centers. The NAKO study received approval from the institutional review boards at all five imaging sites, and all participants provided written informed consent before enrollment [2].

Whole-body MR imaging was performed using a standardized T1-weighted 3D two-point VIBE Dixon sequence acquired in axial orientation across all five imaging sites (slice thickness 3 mm, voxel size 1.41 × 1.41 mm, matrix 320 × 260 × 96, TR 4.36 ms, TE 1.23 and 2.46 ms; 3T MAGNETOM Skyra, Siemens Healthineers, Erlangen, Germany) [3].

For the current study, we utilized MRI data and clinical information from the second data release, which included 30,770 participants who underwent MR imaging between May 27, 2014, and September 30, 2019. We excluded 479 participants due to corrupt or incomplete imaging data and 61 participants with missing MACE observations. To harmonize the age distribution with the UK Biobank cohort, we further excluded participants younger than 40 years (n=6,556), resulting in a final analytical cohort of 23,664 participants with complete whole-body MRI scans available for body composition quantification (**Supplemental Figure 1**). NAKO data were used exclusively to derive sex-specific visceral to subcutaneous adipose tissue (VAT/SAT) ratio thresholds and were not included in survival analyses.

Baseline demographic data available for NAKO participants included age at MRI, sex, height (in m), weight (in kg), waist circumference (WC, in cm), and smoking status. Height and weight were measured using standardized equipment at the imaging centers (Stadiometer 274 for height and medical Body Composition Analyzer 515 for weight, both seca GmbH, Hamburg, Germany). Smoking status was assessed by self-report and dichotomized into ever smokers (current and former smokers) and never smokers, consistent with the classification in the UK Biobank (UKB). Information on prevalent disease and major adverse cardiovascular events (MACE) was assessed to enable receiver operating characteristic (ROC) analysis for determining optimal VAT/SAT ratio thresholds. Baseline characteristics of the NAKO cohort stratified by VAT/SAT ratio are presented in **Supplemental Table 1**. A direct comparison of baseline characteristics between NAKO and UKB participants is provided in **Supplemental Table 2**.

**Statistical analysis**

Unless otherwise specified, all sensitivity analyses were repeated using the fully adjusted model (Model 5 in main manuscript: age, sex, smoking status, WC, and body mass index [BMI]).

***Missing covariate imputation***

Missing covariates were imputed using single random forest-based imputation (1,000 trees; missRanger v2.6.1, R; seed=111). The following variables were imputed: fasting lipids (total cholesterol, low-density lipoprotein [LDL], high-density lipoprotein [HDL], and triglycerides; all in mmol/L), glucose (in mmol/L), HbA1c (in mmol/mol), and systolic and diastolic blood pressure (in mmHg), smoking status, and waist circumference, using age, sex, BMI, VAT and SAT volumes, race/ethnicity, alcohol consumption, history of cancer, antihypertensive medication use, history of hypertension, insulin use, and history of diabetes as predictors. As single imputation does not propagate imputation uncertainty, confidence intervals and p-values should be interpreted accordingly. Cox regressions were repeated in the imputed dataset (n=26,668) using the primary metabolically healthy non-obese (MHN) definition based on International Classification of Diseases (ICD) codes.

***Enhanced metabolic health definition***

To address potential misclassification of metabolic health status, we additionally applied an enhanced MHN definition using the imputed dataset. Diabetes was defined as ICD-coded history, insulin use, HbA1c ≥48 mmol/mol, or fasting glucose ≥7.0 mmol/L. Hypertension was defined as ICD-coded history, antihypertensive medication use, systolic blood pressure ≥140 mmHg, or diastolic blood pressure ≥90 mmHg. The hyperlipidemia definition was unchanged from the primary analysis. Cox regressions were repeated in this enhanced MHN subcohort.

***Alternative anthropometric measures***

Waist-to-hip ratio (WHR) and waist-to-height ratio (WHtR) were derived as WC divided by hip circumference and height, respectively. First, to assess the independent association of VAT/SAT ratio with incident MACE and diabetes after adjustment for alternative anthropometric measures, Cox regressions were repeated replacing WC with WHR or WHtR, while retaining BMI (full model: age, sex, smoking status, BMI, and WHR or WHtR). Second, to assess the incremental prognostic value of VAT/SAT ratio beyond each anthropometric measure, nested Cox models with and without VAT/SAT ratio were compared using likelihood ratio tests and Harrell's C-index, identical to the primary analysis.

***Continuous VAT/SAT ratio and VAT volume analyses***

To assess whether associations were independent of the chosen cutoffs, and to compare the incremental prognostic performance of the VAT/SAT ratio and VAT volume, both were analyzed as continuous predictors. The VAT/SAT ratio and VAT volume showed a right-skewed distribution in both sexes and were therefore log-transformed before analysis. Both variables were standardized to sex-specific z-scores to account for known sex differences in fat distribution [4]. First, Cox regressions were performed separately for each predictor (per 1 standard deviation [SD] of log-transformed sex-specific values) to assess independent associations with incident MACE and diabetes. Second, incremental prognostic value beyond the fully adjusted baseline model was assessed for each predictor using likelihood ratio tests and Harrell's C-index.

***Competing risk analysis***

In the primary analysis, incident MACE and incident diabetes were treated as independent outcomes. To account for the potential influence of competing events, Fine & Gray subdistribution hazard models were fitted for each outcome, with the alternative outcome treated as the competing risk (i.e., diabetes as competing event for MACE analyses, and MACE as competing event for diabetes analyses). A combined follow-up time was defined as the minimum of the MACE and diabetes follow-up times.

***Lag-time and minimum follow-up analyses***

Two sensitivity analyses were performed to assess the robustness of results to early events and short follow-up.

First, to address potential reverse causation, whereby subclinical disease present at the time of imaging may have driven early outcome events, participants who experienced an outcome within the first 1 or 2 years of follow-up were excluded (lag-time analyses). Participants without an event retained their full follow-up time.

Second, to assess whether results were influenced by participants with very short total follow-up, analyses were repeated restricting the cohort to participants with a minimum follow-up of ≥1 and ≥2 years, respectively. Unlike the lag-time analyses, this restriction excluded participants regardless of whether they experienced an event. Both sensitivity analyses were applied separately for incident diabetes and MACE.

***Analysis across metabolic phenotypes***

The primary analysis was restricted to MHN participants. We additionally performed Cox regressions across all four metabolic phenotypes. Participants were classified as metabolically unhealthy by the presence of (1) diabetes, regardless of other metabolic risk factors, or (2) a combination of hypertension and hyperlipidemia. Combined with obesity status (BMI ≥30 kg/m² defined as obese), participants were assigned to one of four phenotypes: metabolically healthy non-obese (MHN), metabolically healthy obese (MHO), metabolically unhealthy non-obese (MUN), and metabolically unhealthy obese (MUO). Participants with prevalent cardiovascular disease were excluded across all phenotypes; for incident diabetes analyses, participants with prevalent diabetes at baseline were additionally excluded. Cox regressions were fitted separately within each phenotype.

***Sex- and age-stratified analyses***

To assess whether the association between VAT/SAT ratio and incident MACE and diabetes differed by sex or age, Cox regressions were fitted separately in males and females, and in participants aged <60 and ≥60 years, respectively. All models used identical specifications to the primary analysis, with sex omitted from models in sex-stratified analyses. Formal tests for effect modification by sex and age were performed by adding a multiplicative interaction term to the pooled model, with significance assessed using likelihood ratio tests.

***Adjustment for race***

Due to the limited number of non-white participants in the UKB imaging subsample, self-reported race was dichotomized into white and non-white. To assess whether results were robust to adjustment for race, analyses were repeated adding this variable as an additional covariate to the fully adjusted primary model.

***Assessment of multicollinearity***

The primary Cox regressions included the VAT/SAT ratio, BMI, and WC in the fully adjusted model. We assessed multicollinearity of anthropometric measures using the variance inflation factor (VIF). VIF requires continuous predictors; the VAT/SAT ratio was entered as a sex-specific log z-score for this assessment. VIF was calculated separately for the MACE and diabetes models. A VIF >5 was considered indicative of problematic multicollinearity [5].

## II SUPPLEMENTAL RESULTS

Baseline characteristics of the NAKO cohort: The NAKO cohort, used for VAT/SAT cutoff derivation, comprised 23,664 participants (mean age 53.9 ± 8.2 years, 44.9% female). 14,876 (62.9%) had a low and 8,788 (37.1%) had a high VAT/SAT ratio. Participants with high VAT/SAT ratios were predominantly male (65.7% vs. 48.8%), older (57.8 ± 8.3 vs. 52.0 ± 7.7 years), had higher BMI (28.1 ± 4.1 vs. 26.3 ± 4.9 kg/m²), larger WC (98.2 ± 11.1 vs. 89.5 ± 13.7 cm), and were more frequently current or former smokers (54.1% vs. 48.7%). Detailed baseline characteristics are presented in **Supplemental Table 1**.

**Comparison between NAKO and UKB cohorts:** Compared to the UKB cohort (N=22,040), NAKO participants N=23,664) were younger (53.9 ± 8.2 vs. 64.6 ± 7.8 years), more frequently male (55.1% vs. 46.7%), had higher BMI (27.0 ± 4.7 vs. 24.4 ± 2.8 kg/m²), larger WC (92.7 ± 13.5 vs. 84.9 ± 10.4 cm), greater VAT (3.35 [interquartile range (IQR) 1.82–5.15] vs. 2.93 [IQR 1.71–4.55] dm³) and SAT volumes (14.41 [IQR 10.95–19.09] vs. 13.85 [IQR 11.01–17.16] dm³), and were more often current or former smokers (50.7% vs. 38.8%). VAT/SAT ratios were similar between cohorts (0.223 [IQR 0.122–0.347] vs. 0.20 [IQR 0.12–0.34]). A detailed comparison is provided in **Supplemental Table 2**.

**Missing covariate imputation**

**MACE:** In the imputed dataset (n=26,668), 378 MACE events (1.42%) occurred. High VAT/SAT ratio remained independently associated with MACE after full adjustment (adjusted hazard ratio [aHR]: 1.27, 95% CI: 1.03–1.58, p=0.029). Adding VAT/SAT ratio to the baseline model resulted in a significant increment in discrimination (C-index 0.685 vs.0.681, LRT p=0.028).

**Diabetes:** 317 incident diabetes events (1.19%) occurred. High VAT/SAT ratio remained independently associated with diabetes (aHR: 1.75, 95% CI: 1.36–2.23, p<0.001). Adding VAT/SAT ratio to the baseline model resulted in a significant increment in discrimination (C-index 0.724 vs. 0.716, p<0.001). Results are presented in **Supplemental Table 3**.

**Enhanced metabolic health definition**

**MACE:** Applying the enhanced MHN definition to the imputed dataset (n=16,177), 154 MACE events (0.95%) occurred. High VAT/SAT ratio remained independently associated with MACE (aHR: 1.73, 95% CI: 1.23–2.43, p=0.002). Adding VAT/SAT ratio to the baseline model resulted in a significant increment in discrimination (C-index 0.734 vs. 0.720, p=0.001).

**Diabetes:** 96 incident diabetes events (0.59%) occurred. High VAT/SAT ratio remained independently associated with diabetes (aHR: 1.85, 95% CI: 1.20–2.85, p=0.005). Adding VAT/SAT ratio to the baseline model resulted in a significant increment in discrimination (C-index 0.754 vs. 0.746, p=0.005). Results are presented in **Supplemental Table 4**.

**Alternative anthropometric measures**

**MACE:** In the model adjusted for WHR, high VAT/SAT ratio was not independently associated with incident MACE (aHR: 1.22, 95% CI: 0.95–1.57, p=0.121), and did not provide incremental prognostic value beyond the baseline model (C-index 0.694 vs. 0.691, p=0.120). After adjustment for WHtR, the association was significant (aHR: 1.28, 95% CI: 1.00–1.63, p=0.050), with modest but statistically significant incremental discrimination improvement (C-index 0.694 vs. 0.690, p=0.049).

**Diabetes:** High VAT/SAT ratio remained independently associated with incident diabetes after adjustment for WHR (aHR: 1.57, 95% CI: 1.18–2.08, p=0.002) and WHtR (aHR: 1.74, 95% CI: 1.32–2.29, p<0.001). Adding VAT/SAT ratio to the baseline model resulted in a modest but statistically significant increment in discrimination beyond both models (WHR: C-index 0.732 vs. 0.727, p=0.002; WHtR: C-index 0.725 vs. 0.718, p<0.001). Results are presented in **Supplemental Table 5**.

**Continuous VAT/SAT ratio analysis and comparison of VAT/SAT ratio vs. VAT volume**

**VAT/SAT ratio**: When analyzed as a continuous predictor (per 1 SD of log-transformed sex-specific values), a higher VAT/SAT ratio was independently associated with increased risk of incident MACE (aHR: 1.17, 95% CI: 1.02–1.34, p=0.022) and diabetes (aHR: 1.50, 95% CI: 1.28–1.75, p<0.001) after full adjustment. Adding VAT/SAT ratio to the baseline model resulted in a statistically significant increment in discrimination for MACE (C-index 0.693 vs. 0.690, p=0.021) and diabetes (C-index 0.726 vs. 0.715, p<0.001).

**VAT volume**: VAT volume was evaluated as an alternative continuous predictor using the same approach. A higher VAT volume was independently associated with increased diabetes risk (aHR: 1.68, 95% CI: 1.31–2.14, p<0.001), while no significant association with MACE was found (aHR: 1.12, 95% CI: 0.93–1.35, p=0.227). VAT volume did not provide incremental prognostic value beyond the baseline model for MACE (C-index 0.691 vs. 0.690, p=0.220), while incremental value was observed for diabetes (C-index 0.723 vs. 0.715, p<0.001).

C-index was marginally higher for VAT/SAT ratio compared to VAT volume for both outcomes. Formal direct comparison between predictors was not performed due to collinearity between VAT volume and VAT/SAT ratio. Results are presented in **Supplemental Table 6**.

**Competing risk analysis**

**MACE:** Accounting for incident diabetes as a competing event, high VAT/SAT ratio remained independently associated with MACE in the fully adjusted Fine & Gray model (subdistribution HR: 1.30, 95% CI: 1.01–1.66, p=0.038).

**Diabetes:** Accounting for incident MACE as a competing event, high VAT/SAT ratio remained independently associated with diabetes in the fully adjusted Fine & Gray model (subdistribution HR: 1.77, 95% CI: 1.35–2.33, p<0.001). Results are presented in **Supplemental Table 7**.

**Lag-time and minimum follow-up analyses**

**MACE:** After exclusion of events occurring within the first 1 and 2 years of follow-up, the association between high VAT/SAT ratio and MACE was attenuated and no longer statistically significant (lag 1 year: aHR 1.28, 95% CI 0.99–1.68, p=0.064; lag 2 years: aHR 1.21, 95% CI 0.89–1.65, p=0.226). Restriction to participants with a minimum follow-up of ≥1 and ≥2 years yielded comparable results.

**Diabetes:** The association between high VAT/SAT ratio and incident diabetes remained significant and strengthened with longer lag periods (lag 1 year: aHR 1.82, 95% CI 1.22–2.86, p<0.001; lag 2 years: aHR 2.44, 95% CI 1.65–3.59, p<0.001). Results were identical when restricting to participants with minimum follow-up of ≥1 and ≥2 years. Results are presented in **Supplemental Table 8**.

**Analysis across metabolic phenotypes**

Of 29,021 participants across all metabolic phenotypes, 22,040 (75.9%) were MHN, 3,376 (11.6%) MHO, 2,435 (8.4%) MUN, and 936 (3.2%) MUO. In diabetes analyses, participants with prevalent diabetes were excluded from MUN and MUO, yielding 1,971 MUN and 670 MUO participants.

**MACE:** MACE event rates varied across phenotypes: 1.3% (297/22,040) in MHN, 1.6% (53/3,376) in MHO, 2.3% (57/2,435) in MUN, and 2.4% (22/936) in MUO. High VAT/SAT ratio was independently associated with MACE in the MHN phenotype only (aHR: 1.30, 95% CI: 1.02–1.66, p=0.037). No significant associations were observed in MHO (aHR: 0.77, 95% CI: 0.44–1.34, p=0.358), MUN (aHR: 0.91, 95% CI: 0.52–1.61, p=0.755), or MUO (aHR: 1.37, 95% CI: 0.55–3.46, p=0.500).

**Diabetes:** Diabetes event rates were 1.1% (251/22,040) in MHN, 4.6% (155/3,376) in MHO, 3.0% (60/1,971) in MUN, and 6.1% (41/670) in MUO. High VAT/SAT ratio was independently associated with incident diabetes in MHN (aHR: 1.77, 95% CI: 1.34–2.33, p<0.001), MUN (aHR: 2.08, 95% CI: 1.07–4.02, p=0.030), and MUO (aHR: 2.38, 95% CI: 1.12–5.07, p=0.024). No significant association was observed in MHO (aHR: 1.19, 95% CI: 0.85–1.66, p=0.306). Results are presented in **Supplemental Figure 2**.

**Sex-stratified analyses**

**MACE:** MACE event rates were 2.0% (202/10,291) in males and 0.8% (95/11,749) in females. In sex-stratified analyses, high VAT/SAT ratio was not significantly associated with MACE in either males (aHR: 1.23, 95% CI: 0.92–1.65, p=0.171) or females (aHR: 1.41, 95% CI: 0.91–2.19, p=0.127). No significant effect modification by sex was detected (p=0.727).

**Diabetes:** Diabetes event rates were 1.7% (173/10,291) in males and 0.7% (78/11,749) in females. High VAT/SAT ratio was independently associated with incident diabetes in males (aHR: 1.83, 95% CI: 1.31–2.58, p<0.001), while the association in females did not reach statistical significance (aHR: 1.54, 95% CI: 0.95–2.51, p=0.080). No significant effect modification by sex was detected (p=0.719). Results are presented in **Supplemental Figure 3**.

**Age-stratified analyses**

**MACE:** MACE event rates were 0.7% (48/6,668) in participants aged <60 years and 1.6% (249/15,372) in those aged ≥60 years. High VAT/SAT ratio was not significantly associated with MACE in either age group (<60 years: aHR: 1.57, 95% CI: 0.85–2.88, p=0.149; ≥60 years: aHR: 1.19, 95% CI: 0.91–1.55, p=0.198). No significant effect modification by age was detected (p=0.246).

**Diabetes:** Diabetes event rates were 0.7% (44/6,668) in participants aged <60 years and 1.3% (207/15,372) in those aged ≥60 years. High VAT/SAT ratio was independently associated with incident diabetes in participants aged ≥60 years (aHR: 1.82, 95% CI: 1.33–2.48, p<0.001), while no significant association was observed in those aged <60 years (aHR: 1.47, 95% CI: 0.78–2.78, p=0.230). No significant effect modification by age was detected (p=0.537). Results are presented in **Supplemental Figure 4**.

**Adjustment for race**

Self-reported race was available for 21,980 of 22,040 participants (99.7%). After additional adjustment for race, high VAT/SAT ratio remained independently associated with both MACE (aHR: 1.30, 95% CI: 1.01–1.66, p=0.038) and incident diabetes (aHR: 1.80, 95% CI: 1.37–2.38, p<0.001). Results are presented in **Supplemental Table 9**.

**Assessment of multicollinearity**

VIF values were below the threshold of 5 for all predictors in both the MACE and diabetes models, indicating no problematic multicollinearity. The highest VIF was observed for WC (MACE: 3.14; diabetes: 2.79), followed by BMI (MACE: 2.16; diabetes: 1.98), reflecting moderate correlation between these anthropometric measures. VIF values for VAT/SAT ratio were 1.19 and 1.13 for MACE and diabetes, respectively.

## III SUPPLEMENTAL TABLES

**Supplemental Table 1: Baseline characteristics stratified by VAT/SAT ratio in the NAKO**

|  | Overall  (N=23,664) | Low VAT/SAT  (N=14,876) | High VAT/SAT  (N=8,788) |
| --- | --- | --- | --- |
| Age (Years) | 53.9 ± 8.2 | 52.0 ± 7.7 | 53.8 ± 8.3 |
| Female sex | 10626 (44.9%) | 7616 (51.2%) | 3010 (34.3%) |
| BMI (kg/m^2^) | 27.0 ± 4.7 | 26.3 ± 4.9 | 28.1 ± 4.1 |
| WC (cm) | 92.7 ± 13.5 | 89.5 ± 13.7 | 98.2 ± 11.1 |
| Current/former smoker | 11995 (50.7%) | 7241 (48.7%) | 4754 (54.1%) |
| SAT (dm^3^) | 14.41 (IQR 10.95-19.09) | 14.24 (IQR 10.57-19.12) | 14.68 (IQR 11.55-19.03) |
| VAT (dm^3^) | 3.35 (IQR 1.82-5.15) | 2.27 (IQR 1.30-3.71) | 5.08 (IQR 3.84-6.53) |
| VAT/SAT ratio | 0.223 (IQR 0.122-0.347) | 0.143 (IQR 0.095-0.269) | 0.383 (IQR 0.210-0.454) |
| Diabetes rate | 6.1% | 3.6% | 10.3% |
| MACE rate | 0.6% | 0.4% | 0.9% |

Values are presented as mean ± SD for normally distributed continuous variables, median (IQR) for skewed continuous variables, and n (%) for categorical variables. BMI, body mass index; IQR, interquartile range; MACE, major adverse cardiovascular events; SAT, subcutaneous adipose tissue; SD, standard deviation; VAT, visceral adipose tissue; VAT/SAT ratio, visceral-to-subcutaneous adipose tissue ratio; WC, waist circumference.

**Supplemental Table 2: Comparison of baseline characteristics between the NAKO and UK Biobank cohorts**

|  | NAKO (N=23,664) | UKB (N=22,040) |
| --- | --- | --- |
| Age (Years) | 53.9 ± 8.2 | 64.6 ± 7.8 |
| Female sex | 10626 (44.9%) | 11749 (53.3%) |
| BMI (kg/m^2^) | 27.0 ± 4.7 | 24.4 ± 2.8 |
| WC (cm) | 92.7 ± 13.5 | 84.9 ± 10.4 |
| Current/former smoker | 11995 (50.7%) | 8559 (38.8%) |
| SAT (dm^3^) | 14.41 (IQR 10.95-19.09) | 13.85 (IQR 11.01-17.16) |
| VAT (dm^3^) | 3.35 (IQR 1.82-5.15) | 2.93 (IQR 1.71-4.55) |
| VAT/SAT ratio | 0.223 (IQR 0.122-0.347) | 0.20 (IQR 0.12-0.34) |

Values are presented as mean ± SD for normally distributed continuous variables, median (IQR) for skewed continuous variables, and n (%) for categorical variables. BMI, body mass index; IQR, interquartile range; MACE, major adverse cardiovascular events; SAT, subcutaneous adipose tissue; SD, standard deviation; VAT, visceral adipose tissue; VAT/SAT ratio, visceral-to-subcutaneous adipose tissue ratio; WC, waist circumference.

**Supplemental Table 3: Missing covariate imputation**

| Analysis | Events | HR (95% CI) | p-value | C-index |
| --- | --- | --- | --- | --- |
| MACE |  |  |  |  |
| Complete case | 297/22040 (1.35%) | 1.30 (1.02-1.66) | 0.037 | 0.694 |
| Imputation | 378/26668 (1.42%) | 1.27 (1.03-1.58) | 0.029 | 0.685 |
| Diabetes |  |  |  |  |
| Complete case | 251/22040 (1.14%) | 1.77 (1.34-2.33) | <0.001 | 0.723 |
| Imputation | 317/26668 (1.19%) | 1.75 (1.36-2.23) | <0.001 | 0.724 |

All models adjusted for age, sex, smoking status, waist circumference, and BMI (Model 5 in main manuscript). Primary analysis: complete case analysis (n=22,040). Imputed cohort: n=26,668 using single random forest-based imputation (1,000 trees; missRanger v2.6.1, R). BMI, body mass index; CI, confidence interval; C-index, Harrell's concordance statistic, a measure of model discrimination (range 0.5–1.0); HR, hazard ratio; MACE, major adverse cardiovascular events; VAT/SAT ratio, visceral-to-subcutaneous adipose tissue ratio.

**Supplemental Table 4: Enhanced metabolic health definition**

| Analysis | Events | HR (95% CI) | p-value | C-index |
| --- | --- | --- | --- | --- |
| MACE |  |  |  |  |
| Primary analysis | 297/22040 (1.35%) | 1.30 (1.02-1.66) | 0.037 | 0.694 |
| Alternative MHN definition | 154/16177 (0.95%) | 1.73 (1.23-2.43) | 0.002 | 0.734 |
| Diabetes |  |  |  |  |
| Primary analysis | 251/22040 (1.14%) | 1.77 (1.34-2.33) | <0.001 | 0.723 |
| Alternative MHN definition | 96/16177 (0.59%) | 1.85 (1.20-2.85) | 0.005 | 0.754 |

All models adjusted for age, sex, smoking status, waist circumference, and BMI (Model 5 in main manuscript). Primary MHN definition: diabetes and hypertension derived from ICD-coded history; hyperlipidemia defined from baseline fasting lipid values; complete case analysis (n=22,040). Enhanced MHN definition: diabetes defined as ICD-coded history, insulin use, HbA1c ≥48 mmol/mol, or fasting glucose ≥7.0 mmol/L; hypertension defined as ICD-coded history, antihypertensive medication use, systolic blood pressure ≥140 mmHg, or diastolic blood pressure ≥90 mmHg; applied in the imputed dataset (n=16,177). BMI, body mass index; CI, confidence interval; C-index, Harrell's concordance statistic, a measure of model discrimination (range 0.5–1.0); HR, hazard ratio; ICD, International Classification of Diseases; MACE, major adverse cardiovascular events; MHN, metabolically healthy non-obese; VAT/SAT ratio, visceral-to-subcutaneous adipose tissue ratio.

**Supplemental Table 5: Alternative anthropometric measures**

| Anthropometric measure | HR (95% CI) | p-value | C-index |
| --- | --- | --- | --- |
| MACE |  |  |  |
| WC | 1.30 (1.02-1.66) | 0.037 | 0.694 |
| WHR | 1.22 (0.95-1.57) | 0.121 | 0.694 |
| WHtR | 1.28 (1.00-1.63) | 0.050 | 0.694 |
| Diabetes |  |  |  |
| WC | 1.77 (1.34-2.33) | <0.001 | 0.723 |
| WHR | 1.57 (1.18-2.08) | 0.002 | 0.732 |
| WHtR | 1.74 (1.32-2.29) | <0.001 | 0.725 |

All models adjusted for age, sex, smoking status, BMI, and the respective anthropometric measure (WC, WHR, or WHtR). HR refers to the association of high vs. low VAT/SAT ratio. BMI, body mass index; CI, confidence interval; C-index, Harrell's concordance statistic, a measure of model discrimination (range 0.5–1.0); HR, hazard ratio; MACE, major adverse cardiovascular events; VAT/SAT ratio, visceral-to-subcutaneous adipose tissue ratio; WHR, waist-to-hip ratio; WHtR, waist-to-height ratio; WC, waist circumference.

**Supplemental Table 6: Comparison of VAT/SAT ratio vs. VAT volume**

| Predictor | HR (95% CI) | p-value | C-index |
| --- | --- | --- | --- |
| MACE |  |  |  |
| VAT | 1.12 (0.93-1.35) | 0.227 | 0.691 |
| VAT/SAT | 1.17 (1.02-1.34) | 0.022 | 0.693 |
| Diabetes |  |  |  |
| VAT | 1.68 (1.31-2.14) | <0.001 | 0.723 |
| VAT/SAT | 1.50 (1.28-1.75) | <0.001 | 0.726 |

All models adjusted for age, sex, smoking status, waist circumference, and BMI (Model 5 in main manuscript). VAT volume and VAT/SAT ratio were each log-transformed and expressed as sex-specific z-scores (per 1 SD increase). Predictors were entered separately in independent models to avoid collinearity. BMI, body mass index; CI, confidence interval; C-index, Harrell's concordance statistic, a measure of model discrimination (range 0.5–1.0); HR, hazard ratio; MACE, major adverse cardiovascular events; SD, standard deviation; VAT, visceral adipose tissue; VAT/SAT ratio, visceral-to-subcutaneous adipose tissue ratio.

**Supplemental Table 7: Competing risk analysis**

| Model | Subdistribution HR (95% CI) | p-value |
| --- | --- | --- |
| MACE (competing event: Diabetes) |  |  |
| Full adjustment | 1.30 (1.01–1.66) | 0.038 |
| Diabetes (competing event: MACE) |  |  |
| Full adjustment | 1.77 (1.35–2.33) | <0.001 |

All models adjusted for age, sex, smoking status, waist circumference, and BMI (Model 5 in main manuscript). Incident diabetes was treated as the competing event for MACE analyses, and incident MACE as the competing event for diabetes analyses. BMI, body mass index; CI, confidence interval; HR, subdistribution hazard ratio; MACE, major adverse cardiovascular events; VAT/SAT ratio, visceral-to-subcutaneous adipose tissue ratio.

**Supplemental Table 8: Lag-time and minimum follow-up analyses**

| Analysis | Events | HR (95% CI) | p-value | C-index |
| --- | --- | --- | --- | --- |
| MACE |  |  |  |  |
| Primary analysis | 297/22040 (1.35%) | 1.30 (1.02-1.66) | 0.037 | 0.694 |
| Lag 1 year | 253/21996 (1.15%) | 1.28 (0.99-1.68) | 0.064 | 0.698 |
| Lag 2 years | 183/21926 (0.83%) | 1.21 (0.89-1.65) | 0.226 | 0.690 |
| Min follow-up ≥1 year | 253/20825 (1.21%) | 1.28 (0.99-1.68) | 0.064 | 0.698 |
| Min follow-up ≥2 years | 183/20485 (0.89%) | 1.21 (0.89-1.65) | 0.226 | 0.690 |
| Diabetes |  |  |  |  |
| Primary analysis | 251/22040 (1.14%) | 1.77 (1.34-2.33) | <0.001 | 0.723 |
| Lag 1 year | 191/21980 (0.87%) | 1.82 (1.22-2.86) | <0.001 | 0.741 |
| Lag 2 years | 144/21933 (0.67%) | 2.44 (1.65-3.59) | <0.001 | 0.768 |
| Min follow-up ≥1 year | 191/20802 (0.92%) | 1.82 (1.22-2.86) | <0.001 | 0.741 |
| Min follow-up ≥2 years | 144/20478 (0.70%) | 2.44 (1.65-3.59) | <0.001 | 0.768 |

All models adjusted for age, sex, smoking status, waist circumference, and BMI (Model 5 in main manuscript). Lag-time analyses excluded participants who experienced an outcome event within the first 1 or 2 years of follow-up. Minimum follow-up analyses restricted the cohort to participants with a total follow-up of ≥1 or ≥2 years regardless of event status. BMI, body mass index; CI, confidence interval; C-index, Harrell's concordance statistic, a measure of model discrimination (range 0.5–1.0); HR, hazard ratio; MACE, major adverse cardiovascular events; VAT/SAT ratio, visceral-to-subcutaneous adipose tissue ratio.

**Supplemental Table 9: Adjustment for race**

| Analysis | Events | HR (95% CI) | p-value | C-index |
| --- | --- | --- | --- | --- |
| MACE |  |  |  |  |
| Primary analysis | 297/22040 (1.35%) | 1.30 (1.02-1.66) | 0.037 | 0.694 |
| Adjusted for race | 297/21980 (1.35%) | 1.30 (1.01-1.66) | 0.038 | 0.694 |
| Diabetes |  |  |  |  |
| Primary analysis | 251/22040 (1.14%) | 1.77 (1.34-2.33) | <0.001 | 0.723 |
| Adjusted for race | 250/21980 (1.14%) | 1.80 (1.37-2.38) | <0.001 | 0.733 |

All models adjusted for age, sex, smoking status, waist circumference, and BMI (Model 5 in main manuscript). Race-adjusted model additionally includes self-reported race (white vs. non-white), which was available for 21,980 of 22,040 participants (99.7%). BMI, body mass index; CI, confidence interval; C-index, Harrell's concordance statistic, a measure of model discrimination (range 0.5–1.0); HR, hazard ratio; MACE, major adverse cardiovascular events; VAT/SAT ratio, visceral-to-subcutaneous adipose tissue ratio.

## IV SUPPLEMENTAL FIGURES


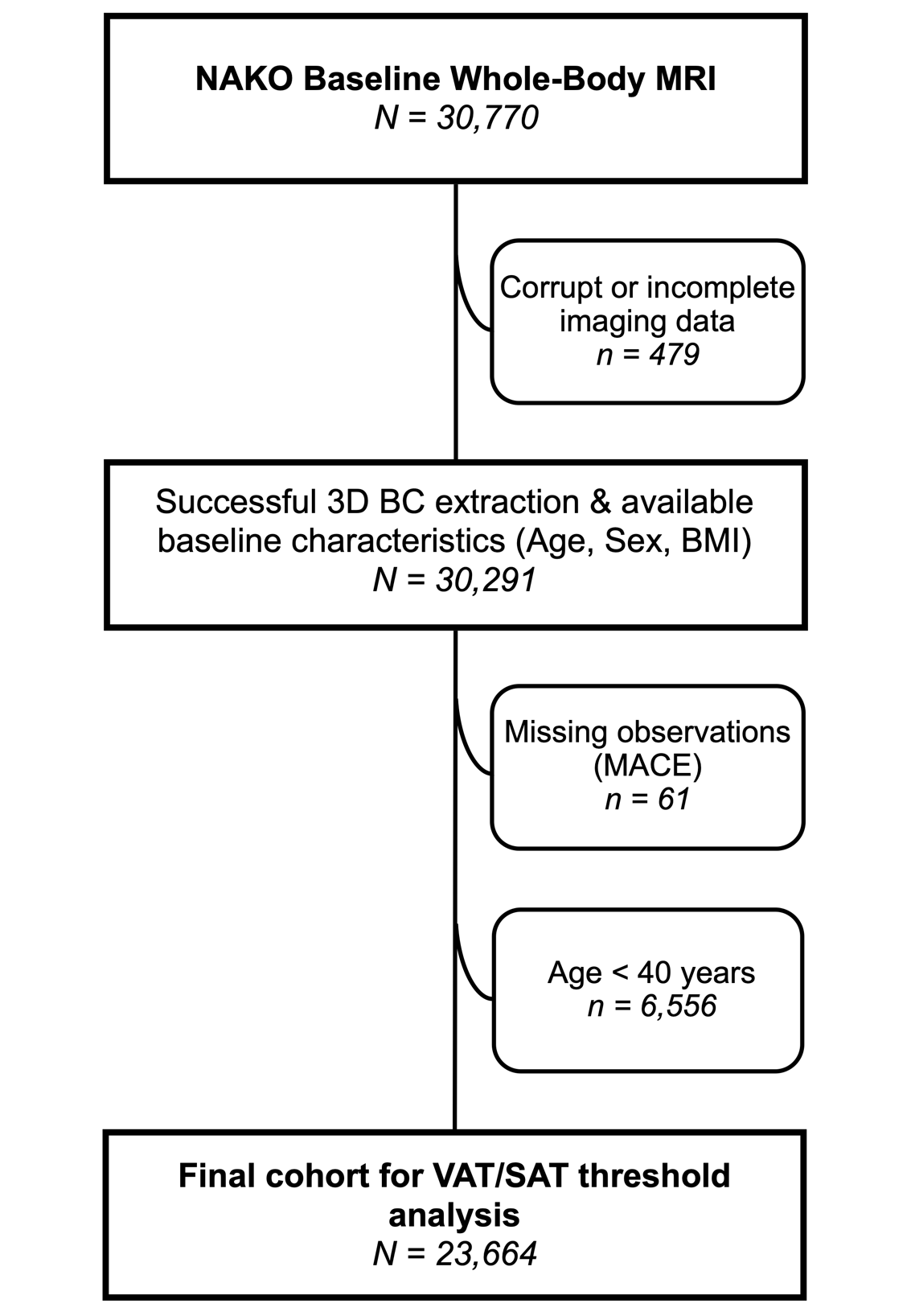


**Supplemental Figure 1: Participant flowchart – NAKO**

Flowchart demonstrating participant selection and exclusion criteria in the NAKO cohort. 3D BC, three-dimensional body composition; BMI, body mass index; MACE, major adverse cardiovascular events; MRI, magnetic resonance imaging; NAKO, German National Cohort; SAT subcutaneous adipose tissue; VAT, visceral adipose tissue.

**Supplemental Figure 2: Association of VAT/SAT ratio and incident MACE and diabetes across metabolic phenotypes.**

Forest plots show hazard ratios from fully adjusted Cox proportional hazards models for (**a**) incident MACE and (**b**) incident diabetes across all four metabolic phenotypes. All models were adjusted for age, sex, smoking status, waist circumference, and BMI (Model 5 in main manuscript). For incident diabetes analyses, participants with prevalent diabetes at baseline were excluded from MUN (n=1,971) and MUO (n=670). BMI, body mass index; HR, hazard ratio; MACE, major adverse cardiovascular events; MHN, metabolically healthy non-obese; MHO, metabolically healthy obese; MUN, metabolically unhealthy non-obese; MUO, metabolically unhealthy obese; SAT, subcutaneous adipose tissue; VAT, visceral adipose tissue.

**Supplemental Figure 3: Associations of VAT/SAT ratio and incident MACE and diabetes stratified by sex.**

Forest plots show hazard ratios from fully adjusted Cox proportional hazards models for (**a**) incident MACE and (**b**) incident diabetes in metabolically healthy non-obese individuals, stratified by sex. Models were adjusted for age, smoking status, waist circumference, and BMI (Model 5 in main manuscript). BMI, body mass index; HR, hazard ratio; MACE, major adverse cardiovascular events; SAT, subcutaneous adipose tissue; VAT, visceral adipose tissue.

**Supplemental Figure 4: Association of VAT/SAT ratio with incident MACE and diabetes stratified by age.**

Forest plots show hazard ratios from fully adjusted Cox proportional hazards models for (**a**) incident MACE and (**b**) incident diabetes in metabolically healthy non-obese individuals stratified by age group (<60 years and ≥60 years). All models were adjusted for age, sex, smoking status, waist circumference, and BMI (Model 5 in main manuscript). BMI, body mass index; HR, hazard ratio; MACE, major adverse cardiovascular events; SAT, subcutaneous adipose tissue; VAT, visceral adipose tissue.

## V SUPPLEMENTAL REFERENCES

1. Peters A, German National Cohort C, Peters A, et al. Framework and baseline examination of the German National Cohort (NAKO). Eur J Epidemiol. 2022;37(10):1107-24.

2. German National Cohort C. The German National Cohort: aims, study design and organization. Eur J Epidemiol. 2014;29(5):371-82.

3. Bamberg F, Kauczor HU, Weckbach S, et al. Whole-Body MR Imaging in the German National Cohort: Rationale, Design, and Technical Background. Radiology. 2015;277(1):206-20.

4. Lumish HS, O'Reilly M, Reilly MP. Sex Differences in Genomic Drivers of Adipose Distribution and Related Cardiometabolic Disorders: Opportunities for Precision Medicine. Arterioscler Thromb Vasc Biol. 2020;40(1):45-60.

5. Vatcheva KP, Lee M, McCormick JB, Rahbar MH. Multicollinearity in Regression Analyses Conducted in Epidemiologic Studies. Epidemiology (Sunnyvale). 2016;6(2).
